# Decision Tool for Herpes B Virus Antiviral Prophylaxis after Macaque-Related Injuries in Research Laboratory Workers

**DOI:** 10.3201/eid2509.190045

**Published:** 2019-09

**Authors:** Sapha Barkati, Hashim Ba Taher, Elizabeth Beauchamp, Cédric P. Yansouni, Brian J. Ward, Michael D. Libman

**Affiliations:** McGill University Health Centre, Montreal, Quebec, Canada (S. Barkati, C.P. Yansouni, B.J. Ward, M.D. Libman);; Sultan Qaboos University Hospital, Muscat, Oman (H.B. Taher);; Hôpital Pierre-Boucher, Longueuil, Quebec (E. Beauchamp)

**Keywords:** herpes B virus infection, B virus, monkey B virus, macacine alphaherpesvirus 1, macaque, antiviral agents, post-exposure prophylaxis, primate workers, research laboratory workers, decision tool, viruses

## Abstract

Macaque-related injuries among primate workers can lead to a potentially fatal B virus encephalomyelitis. We describe a decision tool for evaluating the need for antiviral postexposure prophylaxis and provide a retrospective review of the injuries assessed in our center after its implementation in 2010. Among the injuries studied (n = 251), 40.6% were categorized as high-risk (prophylaxis recommended), 44.2% moderate-risk (consider prophylaxis), and 15.1% low-risk (prophylaxis not recommended). Ten percent of low-risk and 98% of high-risk injuries received prophylaxis (p<0.001). Compared with using universal postexposure prophylaxis, using a decision tool can lead to a standardization of practice and a reduction in prescriptions for antiviral medication.

Herpes B virus or B virus (*Macacine alphaherpesvirus* 1, formerly known as *Cercopithecine herpesvirus* 1) is an alphaherpesvirus that closely resembles human herpes simplex virus (HSV) types 1 and 2 (*1*,*2*). It is endemic in Old World macaques, including rhesus monkeys (*Macaca mulatta*), pigtailed macaques (*Macaca nemestrina*), and cynomolgus monkeys (*Macaca fascicularis*), which are used extensively in biomedical research laboratories (*3*). Infection is acquired primarily through exposure to oral or genital secretions from infected monkeys, with the highest risk of infection occurring during the breeding season in adolescent macaques 2–3 years of age (*4*). The infection in macaques is often asymptomatic, although oral and genital lesions may develop (*5*). Infrequently, B virus infection can lead to disseminated fatal infection in immunosuppressed animals (*3*).

Like HSV in humans, B virus persists in the trigeminal and lumbosacral sensory ganglia of the infected host and can reactivate periodically, resulting in mostly asymptomatic intermittent shedding of the virus in oral and conjunctival mucosa, as well as in genital secretions (*5*). Viremia has been reported in ill macaques but rarely occurs in healthy animals (*6*). Seropositivity among adult macaques (>2.5 years of age) bred in captivity or in the wild can be nearly 100% compared with ≈20% among younger monkeys (*7*). The frequency of viral shedding in seropositive macaques appears to be low, ranging from 2% to 3% in captive macaques at any given time during typical living conditions (*2*,*8*). Factors associated with B virus shedding include immunosuppression, breeding season stress, and a new housing environment (*9*). However, these data should be interpreted with caution given the small number of studies on viral shedding in captive macaques and the focus on rhesus monkeys in most of those studies (*2*).

Human infection with B virus is rare, with >50 documented cases, 21 of which were fatal (*2*,*5*). Moreover, B virus infection has not been documented in humans when macaques are not in captivity. Temples in Asia inhabited by macaques and frequently visited by tourists are sites where macaque-related injuries occur frequently; however, no cases of B virus infection have been reported in these settings (*10*). A case was documented in 1932 in a poliovirus researcher (Dr. W.B. Brebner; hence the name B virus) who was bitten by a rhesus macaque and died of acute ascending myelitis (*3*). Most of the subsequent documented cases reported in the literature occurred in persons who worked with or near macaques (primate workers) (*11*). Documented routes of infection include monkey bites, monkey scratches, injury with contaminated fomites, or exposure of mucous membranes to infectious material from the macaque (*5*). Although the risk for secondary transmission appears to be small, human-to-human transmission of herpes B virus has been documented in 1 case when infection developed in the wife of a man who subsequently died of herpes B virus infection (*12*). In this case, the virus was thought to be transmitted when the wife applied a topical corticosteroid cream to her husband’s vesicular lesions, then to her own contact dermatitis lesions (*12*).

Clinical manifestations in humans usually appear within 5–21 days (range 2 days–5 weeks) of exposure. The virus replicates at the site of inoculation and may initially manifest as nonspecific flu-like symptoms and/or local symptoms at the site of inoculation (itching, tingling, numbness, pain, and vesicular rash). The virus eventually spreads to the central nervous system (CNS) from the upper spinal cord to the brainstem, leading to an acute ascending encephalomyelitis. Patients may also initially have peripheral or CNS symptoms (*13*). There is no cross-protection from HSV 1 and 2 antibodies in humans (*14*). The death rate from untreated infection is estimated to be as high as 70%–80% (*9*). However, it is estimated that 80% of patients survive when treatment with intravenous acyclovir or ganciclovir is initiated promptly (*15*). Certain types of exposures may pose an increased risk of infection. These include deeper, difficult-to-clean wounds (such as needlestick), inadequately cleaned wounds, and wounds closer to the CNS (for example, head and neck) (*5*). Seropositivity to herpes B virus in human primate workers in the absence of disease has not been documented (*16*).

The B Virus Working Group of the Centers for Disease Control and Prevention published recommendations for prevention and treatment of exposure to B virus in 2002, 5 years after the last reported case of a fatal B virus infection in a primate worker (*5*). According to this guideline, antiviral postexposure prophylaxis (PEP) with valacyclovir 1 g 3 times a day (drug of choice) or acyclovir 800 mg 5 times a day for 14 days, within 5 days of exposure, should be recommended or considered for all percutaneous (with loss of skin integrity) or mucosal exposures to potentially infectious macaque tissues or body fluids.

The J.D. MacLean Centre for Tropical Diseases at McGill University has assessed more than 2,000 laboratory workers who have sustained injuries related to accidents while handling macaques over the past 25 years. We designed a decision tool for antiviral prophylaxis against B virus to standardize the approach to injury assessment. In addition, clinicians felt that it would be useful to have criteria for cases in which PEP could be omitted without compromising patient safety. This tool has been used to assess several hundred injuries and, in our hands, it has reduced the rates of antiviral prophylaxis. There have been no instances of viral transmission among our patients, either before or after implementation.

We conducted this study to evaluate the proportion of macaque body fluid exposures for which antiviral prophylaxis was prescribed after the implementation of the decision tool, to describe the characteristics of macaque-related injuries, and to evaluate practitioner compliance with the score-based recommendation for prophylaxis obtained using the decision tool.

## Methods

We conducted a retrospective cohort observational study of macaque-related injuries assessed at the J.D. MacLean Centre for Tropical Diseases during March 2012–August 2016 to assess practice after the implementation of the decision tool in May 2010. We constructed the decision tool after a review of all published cases of human infection with herpes B virus. Risk factors related to the types of exposure were abstracted (Figure).

**Figure:**
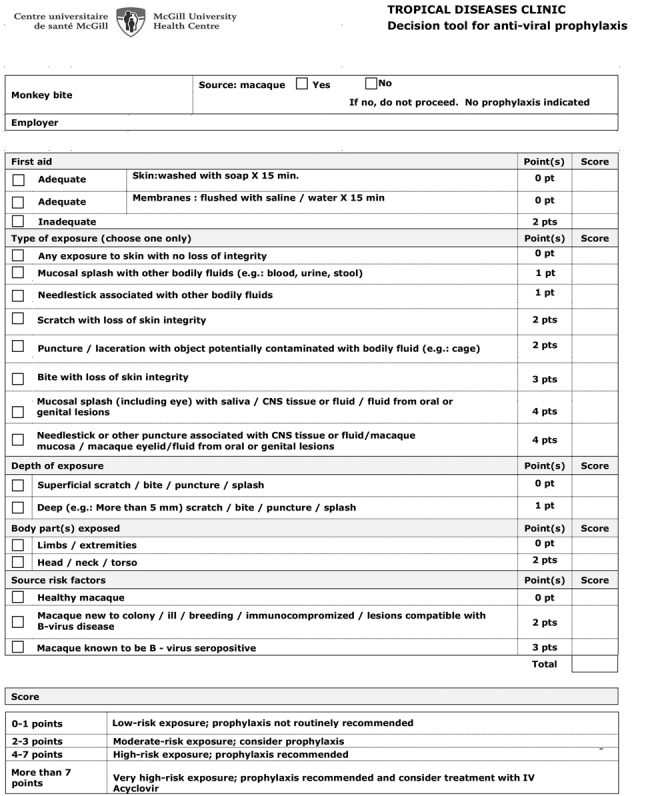
Decision tool used at McGill University Health Centre, Montreal, Canada, for herpes B virus antiviral prophylaxis after macaque monkey–related injuries in research laboratory workers. CNS, central nervous system; IV, intravenous.

For each macaque exposure, 5 major variables were evaluated: the adequacy of first aid for skin and mucous membrane exposure; the type, depth, and location of the exposure; and the characteristics of the source animal. Within the decision tool, each category in the 5 major variables was evaluated on a 4-point scale, from minimal risk (0) to high-risk exposure (4). First aid was considered adequate if the patient promptly washed the skin with detergent for 15 minutes or flushed with saline for 15 minutes after mucous membrane exposure. Injuries associated with the highest risk of infection are exposure with loss of skin integrity, including deep puncture wounds, as well as mucosal exposure associated with potentially infectious specimens (saliva, CNS tissue or fluid, and fluid from oral or genital lesion) or fomites contaminated with such specimens. Exposure of the head, neck, or torso was considered high risk (*5*). The source macaque was also evaluated for risk factors. Exposures to macaques that were either newly introduced to the colony, ill, breeding, immunocompromised, having lesions compatible with herpes B virus, or known to be B virus seropositive were considered high risk. The points were summed over the 5 variables to obtain a final score (maximum score 12). We constructed the scoring system so that all published cases would have received >4 points; this became the threshold for a recommendation to advise prophylaxis. A score of 0–1 resulted in a recommendation against prophylaxis (although no subject was denied prophylaxis if it was demanded). A score of 2–3 was classified as an intermediate score, in which case the practitioner would need to consider PEP based on the individual assessment of risk and discussion with the patient. As part of the macaque-related injury global evaluation, vaccination status against tetanus, as well as the need for antibacterial prophylaxis or surgical intervention, were evaluated.

All cases of macaque-related injury that were treated in our clinic during March 2012–August 2016 were included in the study. Only adults (>18 years of age) who were injured in the course of their work at a research laboratory were eligible. The facilities referring to our clinic were mainly large animal research and testing laboratories. A smaller subset of patients were referred from smaller McGill University–based macaque research laboratories. During this timeframe, no major changes were made to animal handling protocols at any of the referring workplaces. Demographic data, characteristics of injuries (location, type, and depth of exposure), characteristics of the source of the exposure, score and risk categorization according to the decision tool, and antiviral prophylaxis prescriptions were retrieved from computerized patient charts and clinical databases. Descriptive statistical analyses were performed. Categorical variables were expressed as frequencies and percentages and were compared using χ^2^ tests; nonnormally distributed continuous variables were expressed as median and interquartile range. Data were analyzed with MS Excel 2016 (Microsoft, https://www.microsoft.com). The study was approved by the McGill University Health Centre research ethics review board.

## Results

We included a consecutive sample of 251 events (involving 176 individual laboratory workers) during March 2012–August 2016 in the study. The decision tool for antiviral prophylaxis was well received and rapidly adopted by all the physicians in our center. This one-page document allowed a thorough assessment of exposure and categorization of the risk at the same time (Figure).

Table 1 details the demographic and clinical characteristics of the macaque-related injuries. The median age at the time of injury was 32 years (interquartile range 24–38) and 59.1% of the injuries occurred in men. Among all events, 167 (66.5%) patients received antiviral prophylaxis. The first aid was evaluated as inadequate in 14.3% of cases. The most common injury was macaque bite (27%) followed by a scratch with loss of skin integrity (19%); most injuries (74.5%) involved the extremities (Table 2).

**Table 1:** Demographic and clinical characteristics for 251 monkey-related injury events in 176 laboratory workers evaluated during 2012–2016*

Characteristic	Events
Median worker age, y (IQR)	32 (24–38)
Worker sex, no. (%)	
M	149 (59.4)
F	102 (40.6)
Prophylaxis received, no. (%)	
Antiviral	167 (66.5)
Antibacterial	12 (4.8)
Inadequate first aid received, no. (%)	36 (14.3)
Median score (IQR)	2 (1)
Discrepancy between documented and calculated scores, no. (%)	27 (10.8)

*IQR, interquartile range.

**Table 2:** Characteristics of 251 monkey-related injury events by location, depth of injury, and exposure type in 176 laboratory workers evaluated during 2012–2016*

Characteristic	No. (%) events
Location of injury	
Head and neck	51 (20.32)
Limbs	187 (74.50)
Torso	13 (5.18)
Depth of injury	
Deep	39 (15.5)
Superficial	212 (84.5)
Exposure type	
Any exposure to skin with no penetration	12 (4.78)
Scratch with loss of skin integrity	48 (19.12)
Bite with loss of skin integrity	68 (27.09)
Mucosal splash with saliva/CNS tissue or fluid/fluid from oral or genital lesions	6 (2.39)
Mucosal splash with other bodily fluid	15 (5.98)
Needlestick or other puncture associated with CNS tissue/fluid, monkey mucosa/eyelid, or fluid from oral/genital lesions	10 (3.98)
Needlestick or other puncture associated with other bodily fluid	55 (21.91)
Puncture/laceration or with object potentially contaminated with bodily fluid	37 (14.74)

*CNS, central nervous system.

Table 3 describes the categorization of the macaque-related injuries based on the scoring system. Of all injuries, 40.6% were categorized as high risk (prophylaxis recommended), 44.2% were categorized as moderate risk (prophylaxis should be considered), and 15.1% were classified as low risk (prophylaxis not recommended). Among low-risk injuries, 10.5% received prophylaxis, whereas 98% of high-risk injuries received prophylaxis (p<0.001) (Table 4). In the intermediate-risk group, 57.7% received prophylaxis. No case of human herpes B virus infection has occurred in our center, either before or after implementation of this algorithm.

**Table 3:** Categorization of the risk of exposure based on the scoring system for 251 monkey-related injury events in 176 laboratory workers evaluated during 2012–2016

Score category	No. (%) events
Low: 0–1 point	38 (15.14)
Intermediate: 2–3 points	111 (44.22)
High: 4–7 points	102 (40.64)

**Table 4:** Physician compliance with the decision tool for antiviral prophylaxis recommendations for 251 monkey-related injury events in 176 laboratory workers evaluated during 2012–2016

Antiviral prophylaxis	Score category, no. (%) events			Total no. (%) events
	Low	Intermediate	High	
Yes	4 (10.5)	64 (57.7)	100 (98)	167 (66.5)
No	34 (89.5)	47 (42.3)	2 (2)	84 (33.5)
Total	38	111	102	251

## Discussion

We describe a decision tool for evaluating the need for antiviral prophylaxis after a macaque-related injury in research laboratory workers. Because this tool was designed for the assessment of laboratory workers, it may not be relevant for other groups, such as travelers. It is possible that the absence of cases of herpes B disease we observed is related to a low prior probability of infection among the animals in referring research facilities, where veterinary screening programs are in place.

Before the implementation of the tool, our practitioners tended to prescribe antiviral prophylaxis for all macaque-related injuries referred to our clinic. There was a perceived need among both clinicians and employers for standardized criteria that could safely allow for the omission of PEP when the risk was deemed to be negligible. According to current recommendations, the only types of exposure for which antiviral prophylaxis is not routinely recommended are those in which the skin remains intact or when the exposure involves nonmacaque species of nonhuman primates that have never been housed near macaques (*5*). This one-page tool allows practitioners to thoroughly document each exposure and to categorize the risk associated with a given exposure based on a scoring system.

In our study, a sizable number of injuries (44%) fell into the intermediate-risk category; just over half of these were prescribed prophylaxis, despite the fact that no cases of human infection have been reported in patients with this risk profile. The reasons for giving or withholding PEP in this situation was not systematically reported in the patients’ medical charts. In many cases, however, the decision either for or against prophylaxis was actually made by the patient, based on his or her perception of risk versus benefit. As noted earlier, no patient who felt strongly that prophylaxis should be offered was refused.

It has been suggested that the most critical period for the prevention of herpes B virus infection is during the first few minutes after the exposure, and that both the adequacy and timeliness of first aid are essential (*5*). The common recommendation is that wounds be washed with antiseptic detergent (e.g., chlorhexidine) and that mucous membranes should be flushed with water or saline for at least 15 minutes. Review of our cases of macaque-related injuries demonstrated that first aid was inadequate in a number of events (14.3%). A review of cases of herpes B virus infection in humans demonstrated that first aid was inadequate in most cases (*17*).

One fifth of the injuries in our series were located in the head and neck, and ≈6% involved a mucosal splash or needlestick associated with CNS tissues, oral/genital mucosal fluids, or eye fluids from a macaque, all of which are considered high-risk exposures. Herpes B virus moves in the human body along neural pathways in a fashion similar to that of rabies viruses. Studies of rabies viruses have also shown an increased death rate associated with animal bites to the head and neck (*18*). The depth of injury is also associated with an increased death rate in rabies studies. These types of exposures were categorized as high risk for herpes B virus largely based on extrapolation from rabies virus studies (*5*).

Practitioners generally complied with the recommendations of this decision tool. In fact, 98% (100/102) of high-risk injuries received antiviral prophylaxis, whereas 10.5% (4/38) of low-risk injuries received PEP. Arguments in favor of prophylaxis include the fact that B virus infection is highly lethal and that antiviral PEP with acyclovir or ganciclovir has been demonstrated to be effective in prevention of B virus infection in a rabbit model of herpes B infection (*19*,*20*). Although PEP has not been proven to be effective in humans, no cases of herpes B virus infection have been reported in patients receiving PEP within 3 days of exposure (*5*). Arguments against prophylaxis are more numerous, however. A large number of macaque bites and scratches undoubtedly occur each year worldwide, yet documented cases of herpes B infection are very rare (<100), suggesting that transmission is quite inefficient; as noted earlier, the effectiveness of PEP in humans has not been proven conclusively; although they are generally quite well tolerated, side effects such as nausea, headache, vomiting, dizziness, and abdominal pain can occur in subjects taking valacyclovir at the recommended doses (*21*); and antiviral prophylaxis can alter the natural course of and immune response to B virus infection, which may prolong the period of anxiety after the injury and complicate the timing of serologic testing (*8*).

Strict precautions when working with nonhuman primates, adequacy of first aid, and thorough evaluation for PEP form the cornerstone of herpes B infection prevention (*5*,*20*). Education of primate workers regarding the importance of following animal handling protocols and the proper use of personal protective equipment is essential. The number of employees who were injured repeatedly in our series demonstrates problems with nonadherence to, and sometimes the inadequacy of, preventive measures. All these factors emphasize the need for continuous reminders on the protocols to follow in case of an injury.

Our antiviral prophylaxis decision tool allowed a standardized and comprehensive evaluation of macaque-related injuries. Our experience thus far has demonstrated this tool to be well accepted within our clinician community. Based on the literature and our experience to date, the algorithm appears to provide a mechanism for safely withholding antiviral prophylaxis in some cases.
